# Histological and mechanical properties of autologous living tissue biotubes

**DOI:** 10.3892/etm.2013.1040

**Published:** 2013-04-02

**Authors:** XIAO-SONG CHEN, TONG-WEN OU, JIAN ZHANG, JIAN-XIN LI, BING CHEN, HENG-XI YU, YONG-QUAN GU, YE-QING CUI, JING-YAN ZHANG, YAN-LING XU, HAN-CHEN SUN, SHUANG LIU, RONG WANG

**Affiliations:** 1 Departments of Urology, Xuanwu Hospital, Capital Medical University, Beijing 100053, P.R. China; 2 Vascular Surgery, Xuanwu Hospital, Capital Medical University, Beijing 100053, P.R. China; 3 Central Laboratory, Xuanwu Hospital, Capital Medical University, Beijing 100053, P.R. China

**Keywords:** tissue engineering, autologous transplant, small caliber, biotube, collagen

## Abstract

The aim of this study was to explore and evaluate biotubes consisting of autologous tissues. The biotubes were prepared by intra-abdominally embedding silicon rods as moulds. The specimens were analyzed by mechanical tests, histological observation and superficial study. The intra-abdominal implantation of the silicone tubes readily stimulated the development of the biotubes. The biotubes consisted of collagen-rich extracellular matrices. Myofibroblasts appeared as elongated cells with circumferential or longitudinal orientations. Subsequent to one month of embedding, the thickness of the tube wall was 70–250 *μ*m. The burst strength was 1100±187 mmHg and the suturability was excellent. Biotubes that have the ability to be widely variable in their shapes are composed of autologous cells and glomerular extracellular matrices. Biotubes are ideal grafts for tissue engineering as they are able to avoid immunological rejection and are of sufficient mechanical strength.

## Introduction

Peirce (1) and Sparks *et al*(2) independently attempted to apply the wrapping phenomenon to induce the generation of a tubular wrapping tissue as a vascular substitute using an allotransplant implanted in the subcutaneous tissue. However, these attempts failed. All vessels were occluded during early implantation as the lumenal face of this type of graft has directly exposed collagen fibers and eventually promotes thrombosis (3–5).

Tissue engineering technology provides a method for developing novel vascular graft materials. However, existing synthetic and natural materials are not able to meet the demands of this process. Traditional vascular tissue engineering technology uses a polymer material as a stent. However, this polymer material has a mechanical property which makes it difficult to mould. Moreover, its waste products have varying degrees of local toxicity (6). Increasing attention has therefore been awarded to biologically sourced materials. Xenogeneic or allogeneic acellular materials have unique advantages in mechanical strength and compliance. However, the risks of infectious diseases, antigen residues and other problems exist (7,8). Tissue calcification is another significant reason for limiting the application of these materials (9). The collagen-base frame, examined on behalf of recombination extracellular matrix materials, has a traditional material-incomparable advantage in biological compatibility. However, existing problems with mechanical properties and compliance greatly restrict the applications of this type of material in tissue engineering. Several scholars have conducted cross-linking of the collagen-base frame using physical or chemical methods and also by blending the frame with other materials to modify its properties. However, the collagen-base frame remains unsuitable for constructing tissue engineering vessels (7,8,10,11).

An ideal tissue graft should only include a patient’s own cells and extracellular matrix components, as well as having the appropriate mechanical properties and shapes. The tissue materials of the recipients themselves have attracted attention in recent years due to their unique advantages. The wrapping tissue generated by the allotransplant-induced package is a type of cell-free autogenous living tissue material with incomparable biological superiorities beyond those of synthetic materials. In theory, this wrapping tissue completely avoids the rejection reaction and the destructive effect of the non-autograft immune system. This tissue may also be prepared according to the user’s requirements. Therefore, the tissue materials of the recipients themselves are expected to solve the problem of constructing tissue engineering vessels. However, no studies exist on the histological and mechanical properties of such material. The present study investigated the histological components, structure, tensile strength and elongation rate at the breaking point, bursting strength, suture serviceability and other mechanical characteristics of this novel living tissue material.

## Materials and methods

### 

#### Animals

In total, 4 adult female mongrel dogs weighing 20–30 kg were used in the present study. The animals were provided by the Beijing Keyu Experimental Animal Breeding Centre (License No. SCXK 2000-0015; Animal breeding level, grade 1). All procedures were approved by ethics committee of Xuanwu Hospital (Beijing, China).

#### Living tissue preparation

Subsequent to administering mixed anesthesia to the experimental animals via intramuscular injection, the dorsal and abdominal skins were prepared. Each animal was placed in a prone position and the dorsal incision area was disinfected three times with 2% iodophor solution. A surgical knife was used to make two longitudinal small incisions measuring 1 cm each and then two small cavities were formed by conducting blunt separation towards two opposite sides of the subcutaneous tissue. An aseptic medical silicone tube (Jinan silicone factory, Shandong, China) with a length of 4 cm and a diameter of 4 mm was then implanted into each cavity. The incisions were closed with silk sutures. Afterwards, each animal was turned over into the supine position. Following the disinfection of the table covering, an incision measuring 1–2 cm long was made in the abdominal area. A total of 8 aseptic medical silicone tubes were implanted into the abdominal cavities of the animals. The incisions were closed layer by layer. Following one month of post-operative procedures, surgery was performed to completely remove the silicone tube pod that was now wrapped by the peritoneum or subcutaneous tissue. The two ends of the closed pod were cut off following appropriate trimming under sterile conditions. The silicone tubes were taken out to form the final tubular structure.

### Histological testing of living tissue biological tubes

#### Specimen processing

The specimens were removed at one week or one month subsequent to tube implantation and fixed with 10% neutral formalin buffer for 48 h. The specimens were then gradually dehydrated with alcohol, embedded in paraffin wax and cut into slices 4-*μ*m thick.

*Common hematoxylin-eosin (HE) staining.* Subsequent to the specimens being dewaxed with dimethylbenzene, dehydrated with alcohol and stained with HE, the general cell compositions and arrangement distributions of the specimens were observed. The thickness of the living tissue biological tube was measured under an optical microscope.

*Masson staining.* Subsequent to the specimen slices being dewaxed and placed in water, the slices were stained with Masson’s compound staining solution (Fuzhou Wallace New Biological Technology Development Co., Ltd., Fujian, China) for 5 min and washed under running water for 10 min. The specimen slices were then washed with distilled water and soaked in 1% phosphomolybdic acid for 3 min prior to being dried. The slices were soaked in 2% aniline blue solution for 5 min, washed with distilled water, soaked in 1% glacial acetic acid solution for 5 min and conventionally dehydrated and made transparent. Finally, the specimen slices were mounted with neutral gum. Canine natural femoral arteries were collected for the control group.

*Picrosirius red staining.* First, 0.1 g picrosirius red (Sirius Red F3B; Sigma, St. Louis, MO, USA) was dissolved in 100 ml picric acid saturated solution to prepare a picrosirius red solution. The specimens were cut into slices 5-*μ*m thick. The specimen slices were then stained with 0.1% picrosirius red solution for 1 h at 37°C subsequent to being dewaxed and placed in water. The slices were washed under running water for 5 min, made transparent and mounted. Canine natural femoral arteries were also collected for the control group. Observations were conducted using a micropolariscope under darkfield conditions. A closely arranged type I collagen fiber showed strong birefringence and presented yellow, orange and red crude fibers under polarized light. The fiber diameter was larger and the red color was more marked. A type III collagen fiber, arranged in a sparse network shape, presented weak birefringence and green, slim fibers. A type II collagen fiber showed weak birefringence and presented sparse networks with varying colors.

*Observations of the surface structure of the living tissue biological tubes.* The obtained living tissue biological tube was longitudinally split. The inner surface was faced upward, stuck onto dry filter paper, double fixed with 2.5% glutaraldehyde and 1% osmic acid, gradually dehydrated with alcohol, dried at the CO_2_ critical point and sprayed with metal in a vacuum. The ultramicrostructure of the endocoele surface of the biological tube was observed under a scanning electron microscope (SEM; Hitachi S-520, Tokyo, Japan). Canine natural femoral arteries were also collected for the control group. The experimental specimens in the present study were sent to the ultrastructural pathology laboratory of the Affiliated Tiantan Hospital of the Capital Medical University (Beijing, China), for complete ultrastructural observations.

*Immunohistochemical staining of the muscular components of the living tissue biological tubes.* The immunohistochemical method was used to conduct anti-α-smooth muscle actin (anti-α-SMA) and desmin staining to understand the distribution situations of the muscular components in the biological tubes. Immunohistochemical specimens were prepared. The specimens were fixed with neutral formalin, embedded in paraffin wax, cut into slices 4-*μ*m thick, dewaxed with dimethylbenzene, dehydrated with a series of alcohols, repaired with antigen, incubated with 3% H_2_O_2_ at room temperature for 10 min and washed three times with phosphate buffered saline (PBS). Anti-α-SMA antibody (1:100) and anti-desmin antibody (1:100) (Lab Vision-NeoMarkers, Fremont, CA, USA) working liquids were added to the specimens. The mixture was incubated overnight at 4°C and washed three times with PBS. The mixture was then incubated for 20 min and washed with PBS following the addition of agent 1 (polymer helper; Invitrogen, Carlsbad, CA, USA). Agent 2 (polyperoxidase-anti-mouse/rabbit IgG; Zymed) was then added and the mixture was incubated at room temperature for 30 min. The mixture was then washed with PBS, developed with a developer, washed under running water, restained with HE, dehydrated, made transparent and mounted. Canine serum was used to replace the first antibody for the negative control.

### Mechanical testing of the living tissue biological tubes

#### Detection of tensile strength and elongation rate at the breaking point

The specimens were cut into 5-mm wide test strips. The initial length of the tensile machine was set as 2 cm and the specimens were pulled by a Shimadzu AG-5000A Universal Material Testing Machine (Shimadzu, Kyoto, Japan) until they broke. The specimens were frozen and sectioned prior to measurement. The thickness was measured under an optical microscope. Canine natural femoral arteries were also collected for the control group. The measured maximum force was divided by the cross-sectional area of the experimental material to obtain the material tensile strength at the breaking point. The change in the length of the specimen from the beginning of elongation to the breaking point was recorded and divided by the original specimen length to obtain the percentage length change, i.e., the elongation rate of the specimen material at the breaking point.

*Detection of bursting strength.* A living tissue tube specimen was connected to a bursting pressure gauge (Nanjing Wanda instrument and meter plant, Jiangsu, China). The free end was ligatured. The device was filled with PBS solution to soak the specimen. The compression bolt was adjusted to a rate of 50 mmHg/sec at room temperature to increase the pressure in the tube system, which was being recorded by the pressure gauge. Canine natural femoral arteries were used as a positive control in this group. Bursting pressure was defined as the highest pressure in the tube material prior to rupture, representing the tolerance of the material to pressure changes.

*Detection of specimen suture strength.* One end of a specimen was clamped on the tensile machine (Shimadzu AB-I; Shimadzu), while the other end was attached to another fixture of the tensile machine with a 6-0 nylon suture. The distance between the two fixtures was 2 cm. The specimen was pulled at a constant speed of 1 mm/min. The pulling force at the breaking point was recorded. Canine natural femoral arteries were used as the positive control group. The suture strength represented the material suture serviceability.

## Results

### 

#### Biological tube preparation

**Figure 1:**
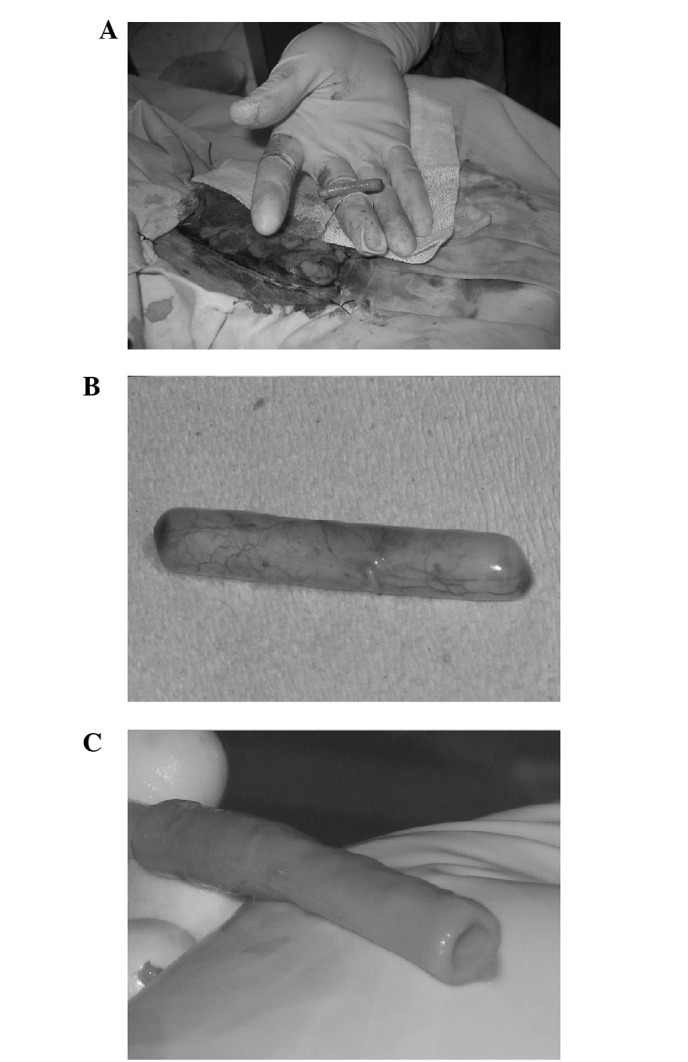
Biological tube preparation. (A) A silicone tube implanted into the abdominal cavity and completely wrapped into the pod capsule-like structure. (B) Enlarged image of a wrapped pod capsule specimen, with a visible small pedicled structure. (C) Fully formed structure of the living tissue biological tube following the trimming of the isolated pod capsule and subsequent to the silicone tube being taken out.

Sampling was conducted one week subsequent to the silicone tubes being implanted into the subcutaneous tissue and abdominal cavity of the experimental animals. The wrapping tissue was thin and the wrapping of the partial silicone tubes in the abdominal cavity was incomplete and fragile. The silicone tubes were therefore not yet suitable for use as *in vitro* materials. After one month, all silicone tubes implanted into the subcutaneous tissue and abdominal cavity of the experimental animals were completely wrapped. The subcutaneous wrapping tissue markedly adhered to the surrounding fascia and was easily damaged during sampling. The majority of the silicone tubes implanted into the abdominal cavity, however, were wrapped in omental tissue (25/32 specimens) and only a few silicone tubes were free in the abdominal cavity (5/32 specimens), thus demonstrating pedicle adhesion with the peritoneal tissue (Fig. 1A and B). In addition, adhesion with the parietal peritoneum was rare (2/32 specimens). When one end of the pod was cut open, no adhesion between the internal silicone tubes and wrapping tissue was observed and the silicone tubes were easily removed. The living tissue biological tubes that were obtained were relatively thick, solid and strong. However, the tubes were pliable in texture, their external surface was covered with a few soft fatty tissues that were easy to remove and the endocoele surface was smooth (Fig. 1C). In addition, all the biological tubes that were obtained were able to better maintain their open status in solution and the endocoele diameter was close to the external diameter of the implanted silicone tubes (∼4 mm). Therefore, the biological tubes implanted into the abdominal cavity for one month were selected as the objects of investigation in the present study.

#### Histological testing of the living tissue biological tubes

**Figure 2:**
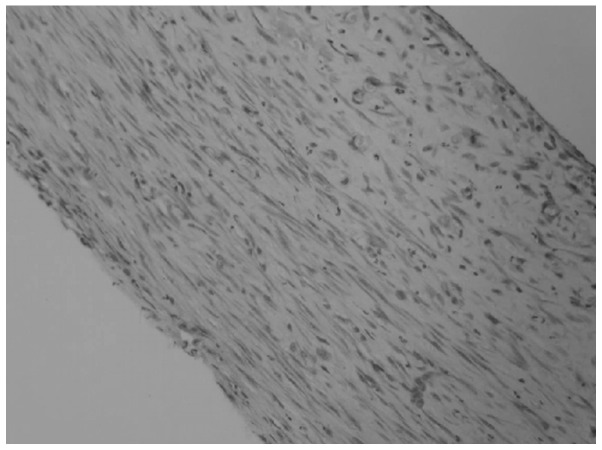
Hematoxylin-eosin (HE) staining of the fresh living tissue biological tube showing the tube wall composed of spindle cells arranged in concentric circles, with a small quantity of inflammatory cell infiltration visible in the tube wall (×400).

Common HE staining was conducted for the slices of the living tissue biological tubes. The wall thickness was nearly uniform under an optical microscope. The tube wall thickness was observed to differ (70–250 *μ*m) between animals or between different sites of the same animal. Histological examination showed that a great number of collagen fibers were arranged in a compact lamellar structure. Spindle cells arranged in concentric circles were visible among them. A small amount of inflammatory cell infiltration was also visible in the tube wall (Fig. 2).

**Figure 3:**
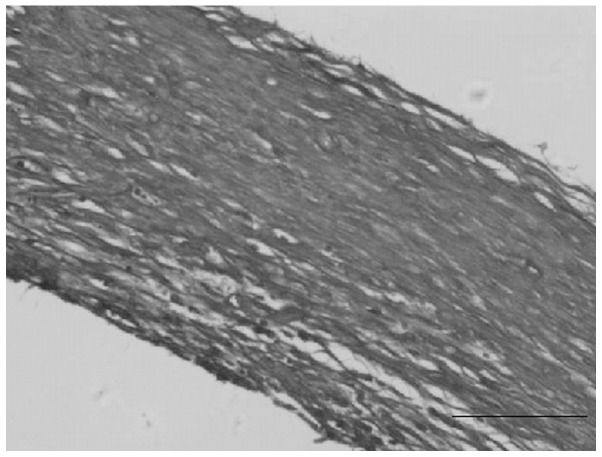
Fresh autogenous living tissue biological tubes. Following the use of Masson’s staining, the collagen fibers were arranged in wave shapes presented as concentric ring-like structures. Smooth muscle-like cells were visible among them (×200).

In the fresh autogenous living tissue biological tubes, the collagen fibers turned blue following Masson’s staining and were arranged in a wave shape, thus presenting concentric ring-like structures. Red-stained, smooth muscle-like cells were visible among them (Fig. 3).

**Figure 4:**
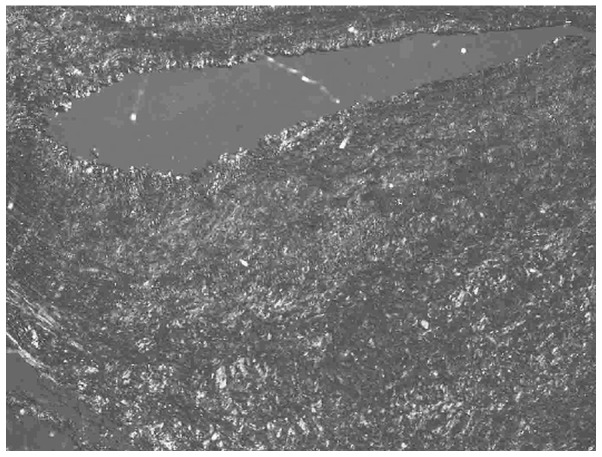
Picrosirius red staining of a fresh living tissue biological tube showing a wide distribution of type III collagen fibers with weak refraction and a scattered distribution of type I collagen fibers with strong refraction (×400).

Micropolariscope observation was conducted under dark-field conditions. The fresh autogenous living tissue biological tube walls showed a wide distribution of type III collagen fibers with green, weak refraction. The scattered distribution of the type I collagen fibers with red-yellow strong refraction was similar to that of the natural blood vessels (Fig. 4).

**Figure 5:**
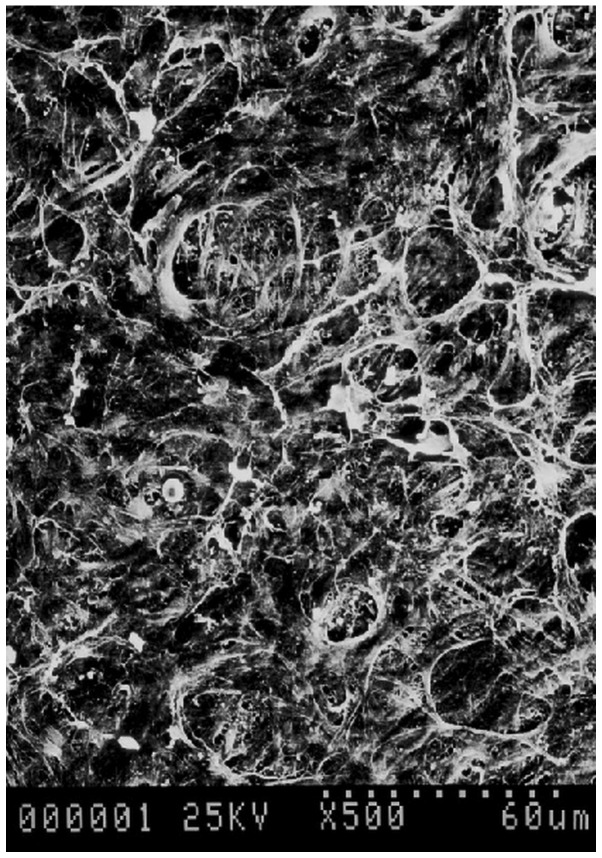
Scanning electron microscope (SEM) observation of the internal surfaces of the autogenous living tissue biological tubes showing a great number of fibrous and membranous extracellular matrix components. Cell bodies and fiber-like apophyses were dimly visible among them (×500).

SEM observation showed a large number of fibrous and membranous extracellular matrix components on the internal surface of the autogenous living tissue biological tubes. The cell bodies and fiber-like apophyses were dimly visible below the matrix. The blood cell components were also occasionally visible (Fig. 5).

**Figure 6:**
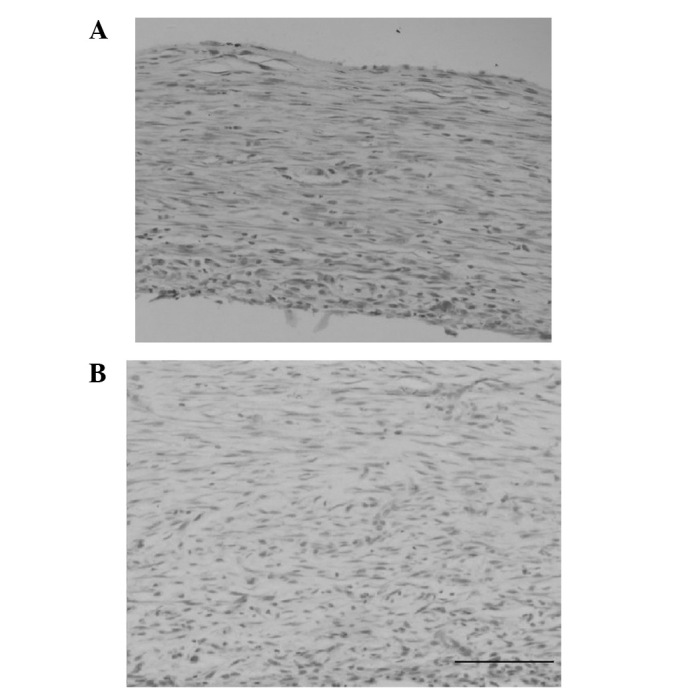
Immunohistochemical staining of fresh living tissue biological tubes. (A) Anti-α-SMA immunohistochemical staining results showing granules and a positive result in the tube wall cells (×200). (B) Anti-desmin immunohistochemical staining results of fresh living tissue biological tube showing negative tube wall cell staining (×400). SMA, smooth muscle actin.

The immunohistochemical study results showed that all the tube walls contained a number of α-SMA-positive cells, whereas desmin staining of the mature smooth muscle cytoskeleton was negative (Fig. 6).

#### Mechanical testing of the living tissue biological tubes

The tensile strength and elongation rate at the breaking point of the living tissue biological tubes were measured to characterize the antitensile capability of the living tissue material in the biological tubes and to represent the material deformability. The tensile strength of the fresh autogenous living tissue biological tubes at the breaking point was observed to be 4.7±2.3 MPa, whereas the elongation rate at the breaking point was 34.2±8.3%. Under the experimental conditions, the tensile strength at the breaking point of the natural femoral arteries was 9.3±3.2 MPa, whereas the elongation rate at the breaking point was 91.5±27.1%.

The bursting strength was measured to determine the maximum internal pressure that the constructed material was able to withstand prior to breaking. This process ascertained if the autogenous living tissue biological tubes had sufficient strength to withstand physical tension. A total of 8 living tissue biological tubes (four groups) from different animals were tested and 4 natural femoral arteries from these corresponding animals were used as the positive controls. The bursting strength of the living tissue biological tubes was 1100±187 mmHg. This bursting strength value was far larger than the internal pressure borne by the natural blood vessels under normal conditions. The pressure tolerance of the natural canine femoral arteries under the experimental conditions was 2280±317 mmHg.

Vascular tissue tolerance to suture tensile force may be defined by the suture tolerance strength. A 6-0 silk suture was used for the tests in the present study. Specimens from the four animals were tested and each specimen was tested three times. The suture tolerance strength of the fresh living biological tubes was observed to be 2.5±0.3 N. The suture tolerance strength of the natural blood vessels was 3.2±0.4 N. The suture tolerance strength values were similar, indicating that the living tissue biological tubes in this group exhibited an adequate suture tolerance strength and would be able to tolerate an end-to-end anastomosis *in vivo* surgery.

## Discussion

The present study attempted to develop a type of autogenous living tissue biomaterial that would be useful for *in vitro* vascular tissue engineering. The proposed material contained only the autogenous cells and extracellular matrix components of the patient and may be used for the *in vitro* construction of tissue-engineered vessels. The tissue wrapping phenomenon induced by an implanted inert allotransplant was applied. First, silicone tubes were implanted into the body of the experimental animals to obtain the pod-like biological tissue formed by wrapping. *In vitro* trimming was then conducted to form a tubular structure called an autogenous living tissue biological tube. The histological components, structure, tensile strength and elongation rate at the breaking point, bursting strength, suture serviceability and other mechanical properties of the proposed material were studied for the preliminary function of wrapping the tubular tissue around a small-caliber artery.

The present study used a hydrophilic silicone tube as a mould and implanted it into the canine body. The pod-like structure wrapped around the implant was formed after one month. The required living tissue biological tube was obtained by *in vitro* trimming. No adhesion existed between the inner surface of the tube and the implant. Implantation of the silicone tubes into subcutaneous tissue was attempted during the preliminary experiment. Similar wrapping tubes were obtained as a result. However, the wrapping tubes were easily damaged during the acquisition process as the wrapping tissue adhered to the subcutaneous fascia. By contrast, the wrapping tubes implanted into the abdominal cavity of the experimental animals were mostly free and their boundaries with the surrounding tissues were clear. Therefore, the present study adopted the method of implanting silicone tubes into the abdominal cavity of the experimental animals. The biological tube walls that were obtained were composed of an extracellular matrix with plenty of collagen fibers and myofibroblasts. The collagen-fiber network was observed as an irregular concentric circle-like structure similar to normal vascular lamellar-like structures (12). The tube wall thickness was 70–250 *μ*m, which is in agreement with that of a biological tube.

The varying common tissues and vascular tissue engineering materials must have appropriate mechanical properties. For example, sufficient strength to withstand a considerable internal pressure without rupturing is required. The proposed material should not bend or tangle due to postural changes and the silicone tubes must be able to serve as sutures and be suitable for use in clinical applications (13). Collagen accounts for 25–33% of the total protein in the human body. Collagen is widely distributed in the extracellular matrix and constitutes tissue organ support. Collagen fibers are closely correlated with the biomechanical properties of tissue organs. In addition, collagen is the basic structural substance of vessels, skeleton, cartilage, muscle tendons, ligaments and other organs. Collagen fibers also cause organs to have a high tensile strength. Histological studies have clearly shown that the static mechanical property of the vasculature is maintained by connective tissues, including collagen fibers. Collagen fibers are distributed in various layers of the tube walls and are located among smooth muscle cells, the subendothelial layer and the adventitia (14,15). The living tissue biological tube constructed in the present study was mainly composed of extracellular matrix components, including collagen tissues (type III collagen was predominant), and is similar to the natural blood vessels (with similar concentric structures). Mechanical study confirms that the prepared living tissue biological tube had an improved antitensile capacity, deformability and adequate bursting strength. The living tissue biological tubes were able to withstand bursting strength values of >1000 mmHg and had surgical suture properties similar to those of the natural arteries. These biological tubes also possessed good mechanical properties.

Several studies have shown that the collagen components of natural arteries are collagen fibers composed of collagen types I and III (16). The spiral directions of the collagen α-chain are contrary to one another. Collagens connect end to end and polymerize in a parallel manner. Collagen fibers exhibit a birefringence phenomenon under a micropolariscope. In addition, the collagen fibers formed by the various types of collagen are inconsistent in thickness. Collagen fibers exhibit a range of colors under a micropolariscope, therefore, estimation of the collagen type is feasible. Picrosirius red is a highly acidic anionic dye that easily reacts with basic groups in the collagen fiber to enhance birefringence and increase resolution. Picrosirius red is a long, unfolded molecule that is able to closely absorb collagen molecules to generate a stable reaction. In addition, this dye does not fade subsequent to staining and it also demonstrates specificity. Thus, picrosirius red is presently the best dye for collagen staining. Collagen types I, II, and III each exhibit various colors under a micropolariscope subsequent to staining. Comparisons between the three collagen types are clear and thus, they may be easily differentiated. Several studies have indicated that, in comparison with current common histological specific and immunohistochemistrical staining, picrosirius red staining has several advantages, including its ease of operation, time-saving qualities, low cost and high specificity and sensitivity (17–19). The living tissue biological tube constructed in the present study was mainly composed of type I and III collagen fibers, which provided adequate strength to the tube.

The extracellular matrix is a type of complete protein molecule secreted outside of the cells following cell synthesis. Matrix components may be generally classified into three categories: glycosaminoglycans/proteoglycans, constructive proteins and adhesive proteins. Studies in recent years have suggested that the *in vivo* extracellular matrix is biologically significant as it is not only a support structure and attachment site for a variety of cells, but it also plays a significant role in the regulation of cellular behaviour and gene expression, the excitation of transmembrane signals and the transformation of cell phenotype and function (20,21). Collagen is a main component of the vascular extracellular matrix. Collagen is located in the subendothelial layer and around the vascular middle-layer of smooth muscle cells (22,23). The living tissue biological tube constructed in the present study was composed of autogenous living cells and natural extracellular matrix components predominantly based on collagen and carrying a number of biological signals. In addition, it is an ideal base-frame material for constructing tissue engineering organs.

The inert allotransplant-induced wrapping phenomenon was applied to implant silicone tubes into the abdominal cavities of the experimental animals for four weeks to construct a type of living tissue biological tube. Histological examination indicated that the living tissue biological tube was composed of autogenous myofibroblasts and extracellular matrix components predominantly based on collagen types I and III. Mechanical testing showed that the biological tube had a good mechanical strength, allowing it to withstand opposing bursting strength values of >1000 mmHg. The biological tube also exhibited a good suture strength. Therefore, the biological tube obtained in the present study may be used as a base-frame material for constructing tissue engineering vessels.
